# A Topological Paradigm for Hippocampal Spatial Map Formation Using Persistent Homology

**DOI:** 10.1371/journal.pcbi.1002581

**Published:** 2012-08-09

**Authors:** Y. Dabaghian, F. Mémoli, L. Frank, G. Carlsson

**Affiliations:** 1 Jan & Dan Duncan Neurological Research Institute, Baylor College of Medicine, Houston, Texas, United States of America; 2 Department of Computational & Applied Mathematics, Rice University, Houston, Texas, United States of America; 3 Department of Mathematics, Stanford University, Palo Alto, California, United States of America; 4 School of Computer Science, The University of Adelaide, Adelaide, Australia; 5 Department of Physiology, Keck Center for Integrative Neuroscience, University of California, San Francisco, California, United States of America; University of Texas Austin, United States of America

## Abstract

An animal's ability to navigate through space rests on its ability to create a mental map of its environment. The hippocampus is the brain region centrally responsible for such maps, and it has been assumed to encode geometric information (distances, angles). Given, however, that hippocampal output consists of patterns of spiking across many neurons, and downstream regions must be able to translate those patterns into accurate information about an animal's spatial environment, we hypothesized that 1) the temporal pattern of neuronal firing, particularly co-firing, is key to decoding spatial information, and 2) since co-firing implies spatial overlap of place fields, a map encoded by co-firing will be based on connectivity and adjacency, i.e., it will be a topological map. Here we test this topological hypothesis with a simple model of hippocampal activity, varying three parameters (firing rate, place field size, and number of neurons) in computer simulations of rat trajectories in three topologically and geometrically distinct test environments. Using a computational algorithm based on recently developed tools from Persistent Homology theory in the field of algebraic topology, we find that the patterns of neuronal co-firing can, in fact, convey topological information about the environment in a biologically realistic length of time. Furthermore, our simulations reveal a “learning region” that highlights the interplay between the parameters in combining to produce hippocampal states that are more or less adept at map formation. For example, within the learning region a lower number of neurons firing can be compensated by adjustments in firing rate or place field size, but beyond a certain point map formation begins to fail. We propose that this learning region provides a coherent theoretical lens through which to view conditions that impair spatial learning by altering place cell firing rates or spatial specificity.

## Author Summary

Our ability to navigate our environments relies on the ability of our brains to form an internal representation of the spaces we're in. The hippocampus plays a central role in forming this internal spatial map, and it is thought that the ensemble of active “place cells” (neurons that are sensitive to location) somehow encode metrical information about the environment, akin to a street map. Several considerations suggested to us, however, that the brain might be more interested in topological information—i.e., connectivity, containment, and adjacency, more akin to a subway map— so we employed new methods in computational topology to estimate how basic properties of neuronal firing affect the time required to form a hippocampal spatial map of three test environments. Our analysis suggests that, in order to encode topological information correctly and in a biologically reasonable amount of time, the hippocampal place cells must operate within certain parameters of neuronal activity that vary with both the geometric and topological properties of the environment. The interplay of these parameters forms a “learning region” in which changes in one parameter can successfully compensate for changes in the others; values beyond the limits of this region, however, impair map formation.

## Introduction

**Figure 1:**
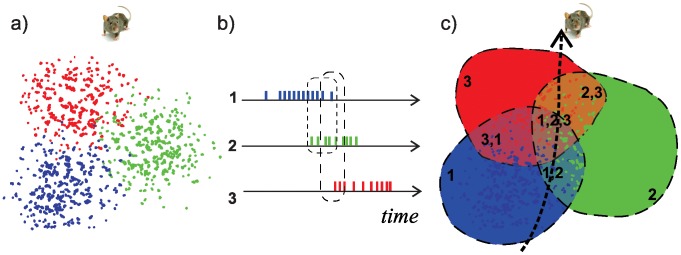
Place fields can be derived from place cell spike trains. (a) As a rat explores a given environment, various place cells will fire in spatially discrete locations. Here, for the sake of simplicity, we depict three place fields as they might arise from spike trains from three place cells, as in the next panel. (b) Schematic representation of spike trains fired from three different place cells as a rat explores an environment. Note that there is contemporaneous spiking activity, or co-firing. (c) The place fields derived from the three place cells in (b): the co-firing patterns indicates areas of overlap of the place fields. When the rat makes a straightforward trajectory through an explored environment, different place cells will be activated and their place fields can overlap.

In order for an animal to be able to navigate a space, remember its route, find shortcuts, and so forth, it must have a fairly sophisticated internal representation of the spatial environment. This internal map is made possible by the activity of pyramidal neurons in the hippocampus known as place cells. Place cells are so named because of their striking spatial selectivity: as an animal (in experiments, typically a rat) explores a given environment, different place cells will fire a series of action potentials in different, discrete regions of the space. Each region, referred to as that cell's “place field,” is defined by the pattern of neuronal firing (most intense at the center and attenuated toward the edges of the field) (**Fig. 1**)—elsewhere, the cell remains silent [1]. The mechanism of this selectivity (why a place cell fires when the rat is here rather than there) is opaque, and how the ensemble of place cells forms a hippocampal map of the environment is only slightly less mysterious. It is believed that the ensemble of place cells activated in a given environment produces a sufficient number of place fields to cover the animal's vicinity [2]: indeed, a rat's path through a small space can later be re-traced with a high degree of accuracy by recording hippocampal spiking activity during its explorations and then analyzing the location, size, and firing rates of a mere 40–50 place fields [3]–[5]. Such experiments suggest that the information contained in place cell firing patterns encodes spatial navigation routes and somehow represents the spatial environment. The hippocampal map thus seems to form the basis of the animal's spatial memory and spatial cognition [6].

How does the brain convert the pattern of neuronal firing into an approximation of the surrounding space? And what information is most important to navigation and spatial memory? In theory, the mental map could represent metric information (distances and angles), affine aspects (colinearity or parallels), or topological information (connectedness, adjacency, containment). The reigning paradigm is that the maps encode geometric information: in fact, most efforts to analyze cognitive maps derived from place fields are based explicitly on the geometry of both the place fields and the environment [2], [4], [6], [7]. But this can not be how the hippocampus or neurons receiving hippocampal output decode place cell firing, because the brain has no direct access to the place fields mapped by neuroscientists. To understand what algorithms the brain might use to decode hippocampal place cell firing, then, we should rely solely on the information provided by place cell spiking activity [8], [9].

If we restrict ourselves to cell spiking activity, the temporal features of the firing pattern become paramount: in particular, if spatial location is the primary determinant of each place cell's firing, then contemporaneous activity or co-firing of several place cells implies that the corresponding place fields overlap. It is, in fact, generally assumed that neurons downstream of the hippocampus interpret place cell spiking patterns based on co-firing. What is not often appreciated, however, is that if place cell co-firing implies spatial overlap of place fields, then the map formed by co-firing is going to be based on connectivity, adjacency and containment—in other words, it will be a topological, rather than a geometric, map.

Indeed, the way place fields cover an environment calls to mind a basic theorem of algebraic topology: if one covers a space *X* with a sufficient number of discrete regions, then it is possible to reconstruct the topology of space *X* from the pattern of the overlaps between the regions [10]. We propose that the hippocampus actually does create a connectivity map derived from place cell co-firing patterns. Although we do not imply any specific interpreting mechanism, we propose nevertheless that it is possible to derive spatial information from place cell firing, with specific implications (and quantifiable predictions) for the qualities of the hippocampal spatial map. We hypothesize that the connectivity map is topological, i.e., any finite structure of overlaps between spatial regions, as represented by temporal overlap of spike trains, can be realized using regions of different shapes or sizes. One implication of this hypothesis is that the information contained in the spike trains is qualitative in nature and can be studied using topological techniques. This is not to deny that the hippocampal connectivity map could contain additional space encoding mechanisms for geometric information (scale, distances, angles)—this question would have to be answered experimentally. Nevertheless, a number of experiments [11]–[14], have demonstrated that smooth geometric variations of the environment produce continuous stretches of the place field layouts that preserve the relative timing between spikes, so that the temporal pattern of spiking remains largely invariant with respect to geometric transformation. This provides some experimental support for our mathematical intuition.

Here we investigate whether a topological connectivity map can be effectively and reliably derived from neuronal spiking patterns using computational tools recently developed in the field of algebraic topology. We show that there exist certain requirements for the firing activity to produce a stable topological map and that the experimentally observed characteristics of firing activity likely satisfy these requirements.

## Results

We will first outline the key concepts underlying our approach; more precise mathematical explanations are provided in the **Methods** section for interested readers.

### Our topological framework: simplicial complexes, spatial and temporal

In algebraic topology, the topological features of a space *X* are defined by its topological invariants, i.e., those properties of the space that are invariant to applied transformations. Topological invariants are described via indices, the simplest of which are the so-called Betti numbers that formalize the counting of loops and holes in various dimensions. The zeroth Betti number, *b_0_(X)*, counts the connected components in the space *X*; *b_1_(X)* gives the number of one-dimensional (1*D*) loops, *b_2_(X)* the number of two-dimensional (2*D*) loops, and so on (see **Methods** and [10]). The Betti numbers can be calculated by an algorithm that analyzes the “cover” of a space *X* by an ensemble of discrete regions [15]. This algorithm uses “nerve of the cover” or “nerve simplicial complex,” *N(X)*, which has as many vertices as there are regions used to cover the space *X*. If two regions overlap, the corresponding vertices, say, *v_i_* and *v_j_*, are considered connected by a 1*D* bond *v_ij_*. If three regions overlap, then three bonds, *v_ij_*, *v_jk_*, and *v_ki_*, support a 2*D* triangular facet, and so on, as the number of overlaps and bonds increase. The complex *N(X)* obtained from a sufficiently dense cover of the space *X* will reproduce the correct topological indices of *X* (see **Methods** for a more precise definition of “sufficiently dense”). The structure of the simplicial complex thus approximates the structure of the environment (see **Methods**).

Drawing on this concept of the nerve simplicial complex *N(X)*, whose structure can be used to deduce the structure of a space, we devised a temporal analogue, the temporal simplicial complex *T* that should give a complete topological description of a space *X* in terms of place cell co-firing. The difference is that the structure of the temporal simplicial complex *T* unfolds over time, *T = T(t)*: as the animal explores its environment and more place cells fire (and co-fire), the structure of the complex *T(t)* grows as *t* increases with the number of spikes. It should thus be possible to trace the emergence of topological information as more and more spikes are fired. When the animal is first introduced to the environment, there would be few data points from place cell spiking, but the data would accumulate as the animal explores, enabling the formation of an internally consistent topological map. Eventually, after a certain minimal time 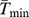, the structure of *T(t)* should saturate, and its topological characteristics should stabilize and produce the correct topological indices, which would indicate the completeness of the topological information.

Given a certain experimental, phenomenological or theoretical description of place cell firing, it should be possible to trace the accumulation of topological information with *t* and discover how much time 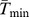 is required to produce the correct topological signature of a particular environment. The temporal framework we propose does impose certain requirements, however: just as there must be a sufficient number of place fields covering a space *X* in order to produce a coherent map of that space, we predict that there are certain conditions that must be met by place cell activity if we are to be able to rely solely on the temporal overlap between neuronal spike trains. First, there should be sufficient co-firing of place cells. Second, the place cells should have sufficient spatial specificity (though there will be a certain amount of biological noise). Third, there should be a realistic learning period in which true signals can be distinguished by their persistence beyond biological noise. These criteria may or may not be met by the hippocampus, given the high variability of biological systems [5], but they follow from our topological hypothesis. In this paper we use newly developed methods from Persistent Homology theory (see **Methods**) to test our hypothesis and to study the temporal dynamics of topological information emerging from the spikes.

### Selecting parameters on which to base the model

The parameters that might be taken into account to define place cell activity are numerous and complex: there are biophysical variables (firing rates, spike amplitude, etc.), behavioral variables (the animal's running speed, etc.), out-of-field firing (not all place cell firing is for spatial encoding purposes), and so forth. For the sake of simplicity, at least for this first attempt to model place cell ensemble behavior, we zeroed in on just a few key parameters that will still enable us to ask key questions.

First we had to decide how to define temporal overlap between spike trains. There is some conjecture in the field that each theta cycle—the basic EEG cycle in the hippocampus, with a frequency of *∼8 Hz*—defines a temporal unit of processing [16], [17]. This suggested to us that we might describe neuronal activity in terms of firing rates, defined over time bins comparable to the theta cycle. Absent any data that speaks directly to this, we defined co-firing as firing that occurred over two consecutive theta cycles.

For this initial analysis, we ignored the details of the spike train structure, such as spike bursting [18] and phase precession [19]–[21] focusing instead on the total number of cells (N), the firing rate (*f*), and a computationally derived place field size (*s*). (In other words, whereas place fields are typically created by mapping recorded neuronal firings onto an actual rat's trajectory through a particular environment, our algorithm ascribes size on the basis of the spread of simulated data points. See **Methods**.)

**Figure 2:**
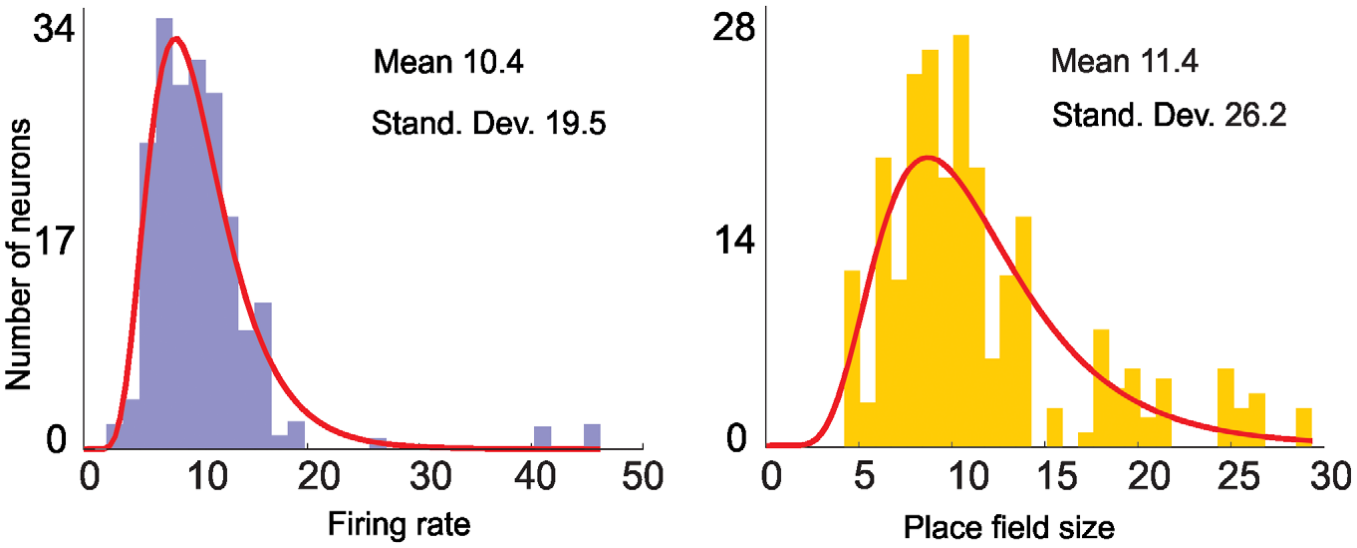
Distributions of firing rate and place field size collected by recording place cell firing as a rat explores a linear track. A typical place cell fires at a rate of ∼10–20 Hz and place fields typically range from 10 to 30 cm across. These experimentally derived distributions serve as realistic constraints on our simulated data by providing proportionality coefficients *a* and *b* so that the shape of the distributions *P*(*f*) and *P(s)* mimics those derived from the experiments. From the data depicted here, *a* = 1.2 and *b* = 1.7.

Thus, for an N-cell ensemble this approach produces 3N independent parameters, *f*_1_, *f*_2_, …, *f_N_*, and *s_x,1_, s_x,2_,…, s_x,N_, s_y,1_, s_y,2_,…, s_y,N_*. Since we are interested in the behavior of the cell ensemble and not just the firing rate of a single place cell or the size of an individual place field, however, we define the values *f_i_* and *s_i_* by the probability distributions *P(f)* and *P(s)*. **Fig. 2** shows a typical shape for these distributions derived from experimental data collected on a linear track (unpublished data, Y.D. and L.F.); these data can be naturally fit by a log-normal distribution with a certain mean and a certain standard deviation, 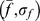 and 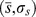, respectively. We further assumed that spiking dynamics are attributable solely to the rat's movement through the environment, i.e., that the probability distributions 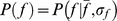, and 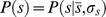 do not depend on time. It is important to note, however, that place fields can be highly plastic during the first few minutes in a new environment [22], so our estimates of the time required to build an accurate topological map are likely to be lower bounds.

Given these starting assumptions and simplifications, the individual firing rates *f_i_* and place field sizes *s_i_* are simply random variables drawn from the stationary distributions *P(f)* and *P(s)*, so the total number N of cells can be treated as another independent parameter characterizing the ensemble. With N included, the activity regime of a place field ensemble is specified by just five parameters: 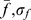, 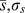 and N. To further reduce the number of parameters in our model, we capitalized on the fact that the mean and the standard deviation of the distributions *P(f)* and *P(s)* can be compared to actual experimental data and are thus not arbitrary (see **Fig. 2**). We can therefore avoid overly broad or overly narrow distributions *P(f)* and *P(s)* by imposing the additional conditions 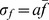 and 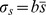, (the higher the individual firing rates and place field sizes found in the ensemble, the wider the spread) and selecting the proportionality coefficients *a* and *b* so that the shape of the distributions *P(f)* and *P(s)* mimics those derived from the experiments. In our computations we used *a* = 1.2 and *b* = 1.7 (as in **Fig. 2**). This last simplification reduces the number of parameters to just three— 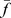, 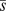, and N —which gives us a 3*D* parameter space that can be readily simulated and visualized.

Given the temporal nature of our map formation model, we will adopt one more simplifying assumption, namely, that all the instances of co-activity that occur between *t* = 0 and 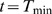 are “remembered” and can be used to establish the structure of the temporal complex *T(t)*. Clearly, any “forgetting” mechanism would cause the temporal complex to deteriorate; information provided by new spike trains could compensate for this loss, but transience of data would increase the map formation time 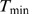.

### The model

The foregoing considerations led to the following (very simplified) working model of place cell activity:

1. Place cell firing activity is a stationary Poisson process described by the rate model [1] (see **Methods**). Theta oscillations, bursting and other effects are not considered.
2. Two cells are considered to be co-active if they fire within two consecutive periods of theta oscillations, i.e., within ∼1/4 sec. We expect shorter time windows would require longer periods for map formation, so this value helps us establish a lower bound on the length of time required to extract connectivity information.
3. The firing rate amplitudes *f_i_* and the computed place field sizes (*s_x,1_*, *s_y,1_*) of the cells in the ensemble are described by independent probability distributions *P*(*μ,σ*), where we used the mode *μ* (to identify the peak of the distribution) and the standard deviation *σ* (e.g., the log-normal or the gamma distributions). In our simulations we used log-normal distributions with 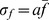 and 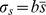, *a* = 1.2 and *b* = 1.7.
4. Retained memory assumption: all firing events occurring up to time *t* can be used in the analysis. We ignore place cell firing that occurs because of the internal dynamics of the hippocampal network (such as reactivation of past experience [23]).

**Figure 3:**
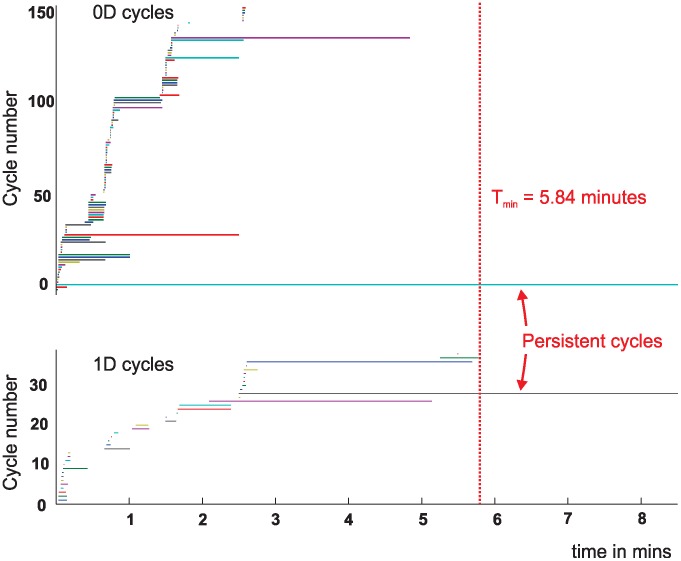
Persistent cycles form a topological barcode. Top and bottom graphs show which 0*D* and 1*D* cycles, respectively, persist in this cell ensemble. Each colored horizontal line represents one 0*D* cycle (top panel) or one 1*D* cycle (bottom panel). Initially, until cells begin co-firing, each 0*D* cycle corresponds to one cell. At later times, both 0*D* and 1*D* cells are emergent phenomena, produced by co-firing of groups of cells. The dotted red vertical line at 5.84 minutes marks the moment when the correct number of loops appears in both 0*D* and in 1*D*, which is the minimal map formation time 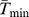. The series of short horizontal bars in both panels (some quite miniscule) and the longer lines that disappear before 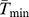 represent topological noise, i.e., cycles that fail to persist. The one persistent 1*D* cycles indicates that the environment in question has one physical (topological) loop, and the single 0*D* cycle indicates that the space is connected, of one piece. Together, this pattern of stable bars forms a barcode that can be ‘scanned’ to discern the topological structure of the environment (see **Methods**).

Our analysis is based on the dynamics of “cycles,” objects that can be used to count the number of topological holes within the temporal complex *T(t)* (**Fig. 3** **,** **Methods**). The intuition informing our approach is that, at early stages of exploration, only a few co-firings will have occurred and so the complex will not adequately represent the topological structure of the environment. As the rat begins to explore an environment at time *t* = 0, the temporal complex will consist mostly of 0-cycles, marking the cells that have fired but not necessarily co-fired. As the rat continues to explore the environment, the co-firing cells will produce links between the vertices of *T(t)*, and higher dimensional cycles will appear. **Fig. 3** shows the cycles in each dimension as a function of time: each horizontal bar represents the timeline of a particular cycle in the complex *T(t)*. At any time *t*, a vertical section will encompass the timelines of all the cycles that are present in *T(t)* at that moment. Once born, a cycle remains stable over a certain period of time, but as *t* increases, most cycles in each dimension will disappear as so much “topological noise,” leaving only a few persisting cycles that express stable topological information. The beauty of this Persistent Homology method [24] (see **Methods**) is that it accommodates such noise so we can distinguish between cycles that persist across time (reflecting real topological characteristics) and transient cycles produced by the rat's behavior (e.g., circling in a particular spot during one trial).

The time required for the correct number of bars (cycles) to appear in every dimension is, by design, the time required to extract the correct topological signature of the environment, which can thus be interpreted as the minimal time 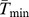 required for the rat to learn the environment. Since this procedure can be applied to various place cell ensembles with different firing profiles, the Persistent Homology method allows us to determine how 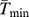 varies with the parameters of hippocampal firing activity. In effect, each set of parameters will produce a “barcode” that can be “scanned” in order to discern the topological structure of the environment.

### Map formation depends on hippocampal state

**Figure 4:**
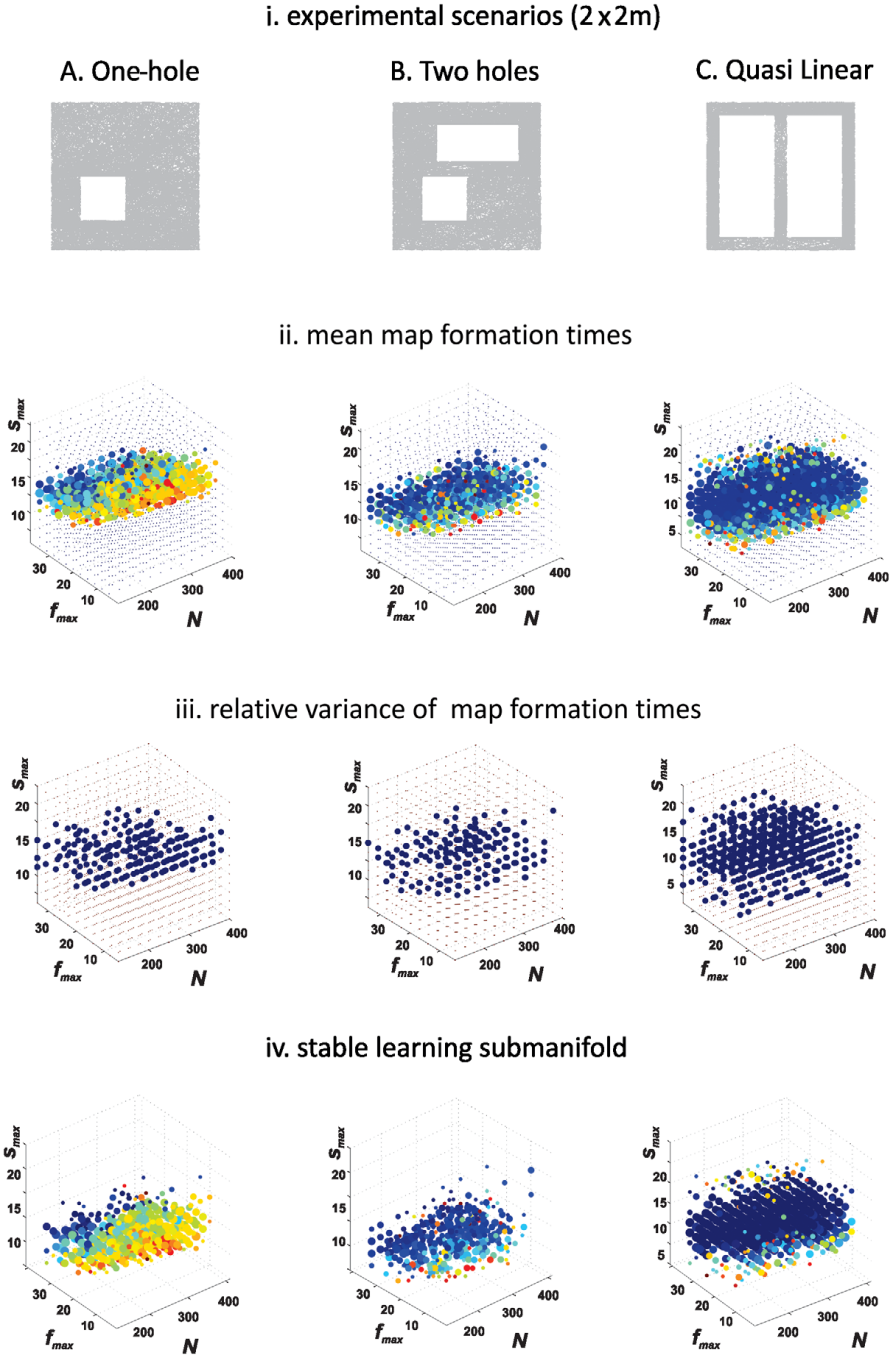
Variations in topology place different demands on hippocampal state. The *top row* depicts three experimental configurations, each two meters square, for our computational simulations; note that the second and third scenarios (B and C) are topologically equivalent but geometrically different, and that scenario C will force our simulated rat to adopt a quasi-linear trajectory. The dense network of gray lines represents the simulated trajectories. *Second row*: Point cloud approximations that reveal mean map formation times for each space configuration. Each dot represents a hippocampal state as defined by the three parameters (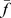, 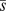, and N); the size of the dot reflects the proportion of trials in which a given set of parameters produced the correct outcome; the color of the dot is the mean time over ten simulations. If, for example, one set of parameters produced the correct topological information in 6 out of 10 trials, the dot will be 60% of the size of the largest dot, and the color will reflect the mean map formation time for the correct trials. (Blue represents success within the first 25% of the total time; green within the first 50%, yellow-orange within the first 75%, and red means success took nearly the whole time period. The maximal observed time was 4.3 minutes for configuration A, 11.7 minutes for B, and 9.3 minutes for C.) Note how the third scenario (C) contains a preponderance of blue dots, indicating that the majority of hippocampal states easily mapped this environment. This is because the two holes are so large that a rat is virtually forced into a straight-line trajectory. *Third row:* Each dot represents the relative standard deviation of map formation times 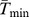 for successful trials where 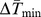 is small (<0.3). *Fourth row*: Combining the mean map formation times (second row) with the robustness requirement 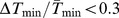 (third row) reveals a domain of stable, robust map formation times that we call the core of the region *L* in the text.

We simulated map formation times using different place cell parameters and three separate planar 2×2 meter areas with 1 or 2 holes (**Fig. 4** **, top row**). (We chose this size because it is similar to that of experimental spaces used for neuroscientific studies of rat place cell properties.) The topological features to be detected are the number of holes in each environment. For the 2-hole environments we considered two cases with different hole sizes to vary the geometrical characteristics of the environment while keeping its topology fixed.

The trajectories were simulated to be: 1) sufficiently ergodic to represent non-preferential exploratory spatial behavior (i.e., there was no artificial circling or other ad hoc favoring of one segment of the environment over another). The spatial occupancy of the immediate vicinities of the holes and of the corners was therefore higher than the average, which is similar to patterns of spatial occupancy in the open field and linear track experiments. 2) The mean and the maximal speed were kept within the range of typical experimental values (based on our experience; the mean speed was chosen to be slightly higher than a typical experimental mean value in order to get a lower estimate for the learning time 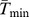). Lastly, 3) the distribution of the moment-to-moment changes in the direction of the simulated rat's movement, Δ*φ_sim_*, matches the experimental histogram of Δ*φ_exp_*.

We asked whether, and for which ensembles, the place cell spiking signals would be able to produce a temporal simplicial complex with the correct number of topological loops (Betti numbers; see **Methods**) in every dimension—the connectivity of the space (0*D* loops) and the correct number of 1*D* loops. In each of these environments we simulated a set of 1000 place cell ensembles by independently varying three parameters of neuronal activity. We probed ten distributions of firing rates, with the mean maximal rate, 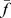, ranging from 2 to 40 Hz, and ten distributions for the place field sizes with 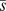, ranging between ∼10 and ∼90 cm. The size of the population varied independently from N = 50 to N = 400 cells. In each case, the centers of the place fields were scattered randomly and uniformly over the environment. For each combination of the parameters 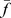, 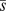, and N —which we can say defines a hippocampal ‘state’—the computation was repeated 10 times (total 10×10×10×10 = 10,000 ensemble simulations), which allowed us to compute the average time 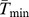 required for the emergence of the correct topological signature for each specific choice of the ensemble parameters, 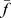, 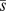, and N. Although the simulated trajectory was fixed, we chose a new set of place field centers for each set of 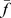, 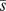, and N for each repetition.

The results are shown in **Fig. 4**. The three panels across the second row show the mean map formation times 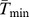 (in minutes) for each of the three environments in the top row. Each point on this diagram represents a particular place cell ensemble with a certain mean ensemble firing rate 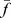, mean place field size 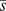 and number of cells N. The sizes of the dots represent the percentage of repetitions in which a given set of parameters (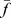, 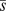, and N) produced the correct outcome: the largest dots correspond to the most successful ensembles, and the sizes of the smaller dots represent the percentage of trials producing the correct outcome for that set of parameters. The color of the dots represents the value of the mean map formation time 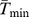 (see Figure legend). Some ensembles consistently produced the correct topological signature for all 10 repetitions, even in very short time frames (large blue dots), whereas other ensembles either produced the correct signature in only a fraction of repetitions (smaller dots) or repeatedly failed to produce the correct result over long periods of time (smallest red dots; 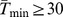 mins, i.e., ∼20,000 theta cycles).

**Figure 5:**
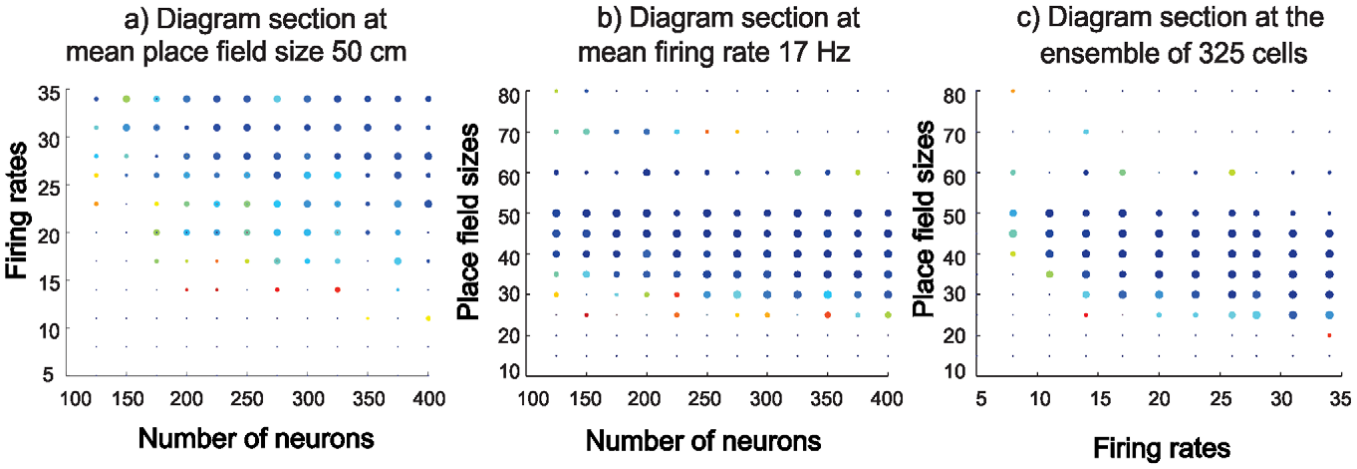
2D sections highlight dependence of map formation times on hippocampal state. These 2D sections are based on the point cloud data in **Fig. 4C**, second row (far right). Dot sizes and colors represent the same characteristics as described in **Fig. 4** (i.e., the larger and bluer the dot, the more successful and more rapid the map formation). **Graph A** fixes the mean place field size at 50 cm, and shows that robust map formation in this case requires a larger number of cells firing at a higher rate. **Graph B** shows that, at a mean firing rate of 17 Hz, any ensemble size between 100 and 400 neurons can fairly rapidly form a correct topological map as long as the place fields are between 50 and 80 cm. **Graph C** shows that an ensemble of 325 cells can have mean firing rates from 10 to 35 Hz and form maps quickly and accurately with place field sizes from 40–80 cm. In short, smaller place cell ensembles, with low mean firing rates (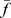
<10 Hz) and too small (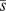
<20 *cm*) or too large (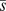
>100 *cm*) mean place field sizes, fail to produce the correct topological signature. In contrast, sufficiently large place cell ensembles with higher firing rate neurons and well-tuned place fields reliably capture the topological structure of the environment in a time frame comparable to the experimentally observed map formation period.

These data illustrate, first, that the firing activity of smaller place cell ensembles (*N≤150*), characterized by low mean firing rates (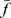
≤10 Hz) and by small mean place field sizes (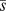
≤20 *cm*), consistently failed to produce the correct topological characteristics of the environment. Similarly, ensembles with very large place field sizes (low spatial selectivity of the place cell's firing, high 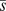 values) also failed to produce the correct topological signature. Both types of cases are represented by the dots on the periphery of each cloud (**Fig. 4** **, second row**). In contrast, larger place cell ensembles with higher firing rates and well-tuned place fields reliably captured the topological structure of the environment within 2–5 mins. As a result, each point cloud can be conceived as containing within its fuzzy boundaries a learning region *L*: a submanifold in the space encompassing the hippocampal states that produce the correct topological map within a biologically plausible time-frame. This pattern is clarified by the 2*D* sections of the 3*D* diagram (**Fig. 5**, **S1** and **S2**).

It is noteworthy that the points with intermediate sizes, representing the partially failing ensembles, tend to diffuse out from the center of *L* to the sparser boundary region of the cloud. This neatly illustrates the transition that occurs between the hippocampal states that consistently produce stable, topologically accurate maps (interior points of *L*), and those that do not (dots outside of *L*). Thus, for all hippocampal states within *L*, the 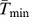 values show an orderly, regular dependence on all three variables 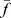, 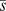, and N. Despite the stochastic nature of the model, then, the minimal map formation time 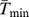 can be approximated by a well-defined, continuous function of the parameters, 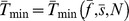. If the firing activity regime moves out of *L*, then the time 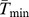 abruptly increases at the boundary of this region.

### The map formation region is stable and robust

**Figure 6:**
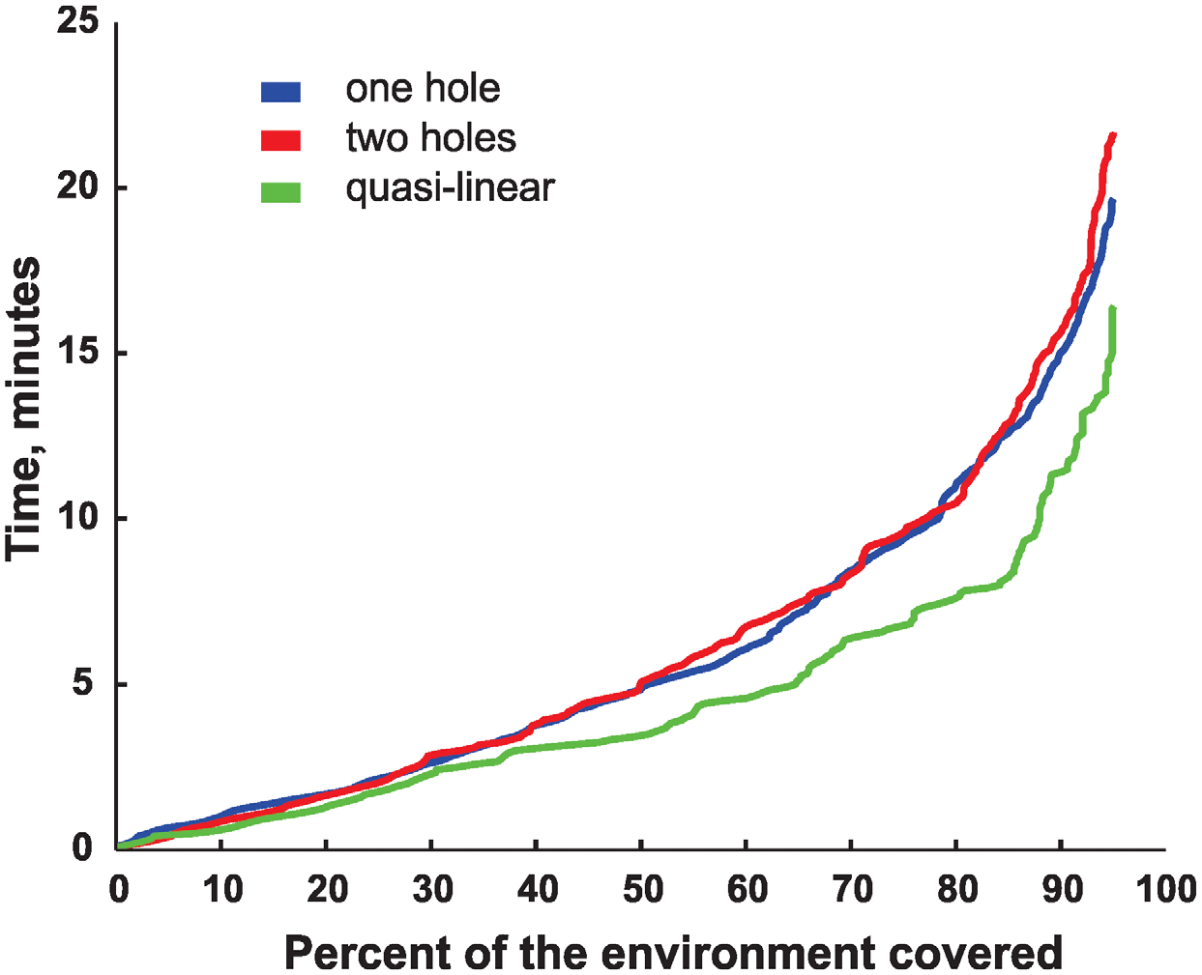
Ergodicity times for the three environments shown in **Figure 4**. For each environment, the graph shows how much time is required to cover a certain percentage of the 3×3 cm spatial bins. This ergodic time scale shows that it takes approximately ten minutes for a rat to cover 80% of the environment; by comparison, the topological map formation time for stable regimes is much lower.

It is noteworthy that at the core of *L*, the characteristic minimal map formation time is 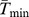
≈2–5 mins, which is comparable to the biological learning time in rats and mice in simple environments [25], [26]. Indeed, the characteristic time 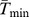 is shorter than the time it takes the trajectories themselves to cover environments A, B, and C (see **Fig. 6**); in other words, the topological model forms maps more rapidly than simply computationally covering the simulated space. As noted above, one of the key hypotheses of our model is that map formation time 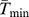 is included in the biological learning time. We reasoned that before the “topological noise” stabilizes, it is not possible to tell how many correct loops there will be or which ones are going to persist (see **Fig. 3**), so that prior to 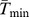 the spatial information encoded by place cell firing is unstable and probably incomplete. Therefore, if the spatial map produced by hippocampal activity is based on interpreting the co-firing patterns, one of the main qualitative predictions of this approach is that the biological learning time can be estimated by 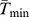. If, for example, the map formation time for a place cell ensemble in Rat A is 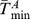, and for a place cell ensemble in Rat B is 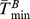, and 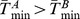 then Rat A will take longer to learn an environment than Rat B. This difference should be observed in the Morris water maze task and other behavioral experiments.

**Figure 4** also shows that the size of the learning region *L* depends on the complexity of the environment. *L* is largest for environment C, in which topological connectivity is defined by the quasi-linear order of place cell firing. The region *L* for environment B is the most compact, reflecting the fact that this environment is topologically the most complex because the navigational paths are indexed by two topological indices (defined by the fundamental group π_1_), so that two persistent loops have to be extracted from a set of non-persisting loops. At the same time, it is also more geometrically complex than the quasi-linear environment (**C**), because it allows 2*D* motion.

It is also important to note that the mean map formation time 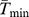 produces a stable, robust core of the learning region *L*. To characterize the stability of 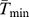 values, we considered the standard deviation of the minimal map formation times, 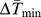 computed for each fixed value of 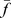, 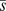, and N. The third row of panels in **Fig. 4** demonstrates the relative standard deviation of the map formation times, 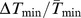 as a function of 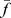, 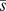, and N. The variations in map formation times increase towards the boundary of the learning region, i.e., the place cell ensembles defined by parameters at the boundary of *L* are successful only a fraction of the time—these ensembles are “unstable” in their map formation ability, or we might say the maps themselves are unstable. (One can imagine a rat with some impairment being unable to learn a space because its mean place cell firing rate is a little too low to produce consistent information about the test environment.) Inside *L*, the values of 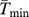 vary less. To emphasize this, the subregion 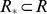 of points 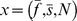 in which the relative standard deviation of the 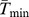 values is less than 30% (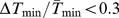) (**Fig. 4** **, third row**).

Finally, in order to single out the hippocampal states for which the mean map formation time 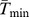 is not only biologically plausible but also robust, we combined the requirements 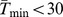 mins (used to build **Fig. 4ii**) and 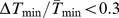. The resulting robust learning region at the core of *L* (**Fig. 4** **iv**) shows that the parameters of the place cell ensemble that guarantee reliable map formation form a well-defined submanifold in the parameter space. This submanifold can be computed using the methods outlined above for each specific environment and for each specific model of neuronal firing, allowing us to relate the geometric and the topological features of the space with the biological parameters of hippocampal place cell activity.

In summary, it is usually assumed that an ensemble of cells with spatially selective firing will naturally encode a spatial map. Our results demonstrate that the spatial selectivity of firing does not, by itself, guarantee a reliable mapping of the actual environment. The geometric shape of the learning region ***L*** and the distribution of the 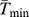 values within ***L*** depend on the global geometry and topology of the environment. This means that place cells cannot be ‘agnostic’ about the scope and nature of the spatial encoding task: the geometry of the environment sets limits on the parameters of neuronal activity that are able to lead to a coherent topological map. Despite the stochastic nature of the system, well-defined mean map formation times 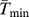 not only exist inside of the stable learning region ***L***, but their values can be approximated by a continuous function of the place cell ensemble statistics. The latter implies that a continuous variation of the hippocampal state within ***L*** will result in a continuous change of the mean map formation time value 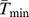. The hippocampus can thus change its operating state inside ***L*** without compromising the integrity of the topological map, such that the size and the shape of ***L*** reflect the scope of the biological variability that the hippocampus can afford in a given environment. The larger the region ***L***, the more stable the map.

## Discussion

We have examined the dynamics of hippocampal spatial map formation beginning with arbitrary place cell activity regimes, both those that resemble biological cells and those that do not. We created a computational program to simulate map formation with three independent variables: the firing rate of the place cells, the size of the place field, and the number of cells. We then tested the model on three different scenarios (which included two topological configurations and two different geometries), and repeated the simulation in each scenario 10 times prior to statistical analysis. Our simulations show that in order to form a reliable topological map of the environment, the place cell ensemble must operate within certain parameters—outside these parameters, place cells can be spatially specific but will not be able to produce a reliable map. It is noteworthy that the parameters for place cell firing and place field size that produced a robust map formation region ***L*** correspond well with experimentally observed place cell firing rates and place field sizes: when the simulated place cells fired at rates either below or above a certain range, or when the simulated place field sizes fell above or below a certain range, what we call the learning region failed to form (**Figure 5** **, S1 and S2**). Mathematically, the model could have required any set of values to work: firing rates of 500 Hz and place field sizes of 2 cm, for example. There was no *a priori* reason that the parameters should fall so neatly into biological range. The fact that they do lends support to our topological paradigm, despite the simplicity of this first model.

### Other parameters and models of place cell behavior

Our current model relies on a simple spike train structure based on a Gaussian firing rate (**Methods**). We ignored many biological parameters of place cell activity, such as synaptic connections, theta phase procession and spike bursting, and out-of-field firing. The ensembles we used were also rather small, ∼400 neurons, which is less than 1% of the number of cells that are believed to be active in a rat's hippocampus during its exploration of a new environment [27] and about 2% of that number for mice [28]. Larger numbers of cells can be incorporated into future versions of the model, which will lead to a more realistic description of the hippocampal spatial map. Although we expect that the quantitative predictions of the model will change as more subtle neurophysiological phenomena are included, we do not anticipate that the overall structure outlined in the current, basic model will change qualitatively. For example, preliminary analyses suggest that the phenomenon of theta precession and multiply connected place fields affect map formation time, i.e., the size and the shape of region ***L*** *_model_* but do not change the fact of ***L***'s existence or the existence of the function 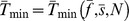.

One could conceivably choose any valid set of parameters to define hippocampal states that produce a model-defined learning region *L_model_*. The result will correspond to the actual, biological place cell map only to the extent that the starting model accurately captures relevant aspects of place cell activity. For example, the Continuous Attractor Neural Network Models [29], which includes (among other things) synaptic efficacies, could be tested for the topological completeness and robustness of the map that it produces. In the absence of exact knowledge about place cell activity in a specific animal, the structure of ***L_bio_*** can be studied using statistically defined (experimental or model-generated) characteristics of neuronal activity. The approach we have outlined here is thus one means of testing the efficiency of other place cell activity models in forming spatial maps.

The topological model predicts that: (1) the parameters describing the hippocampal place cell map in healthy animals should fall inside of the stable learning region ***L*** computed for the given environment, and (2) the hippocampal state might drift towards the boundary of stability or even leave the stability region as a result of a deterioration of neuronal activity. As long as the parameters used in the model are phenomenological characteristics of neuronal firing, the structure of the learning region ***L*** *_model_* will define the effect that a particular parameter has on the hippocampal place cell map as a whole. With this approach, there is no need for *a priori* assumptions about place cell firing rates or the parameters that define place field sizes. Instead, the correct values can be estimated based on the structure of the computed stability region. The fact that the values typically observed in electrophysiological recording experiments fall within the region of stability shown in **Fig. 4** is a testament to the validity of the grounding assumptions of our model as outlined above.

### Implications of the topological model for spatial learning

Despite its simplifications, the current model allows us to examine whether a particular set of place cell parameters can be used to map a given environment and vice versa, and to reason about the effect of the geometry and topology of an environment on place cell behavior. For example, **Figure 4** demonstrates that greater topological complexity reduces the size of the stable learning region ***L*** by constraining the range of hippocampal states capable of forming accurate maps in a reasonable amount of time. This is, in fact, what is observed in experiments that include a large number of objects (enriched environments) and are thus more geometrically and topologically complex than the standard environments: the firing rates and the number of active cells tend to increase [30], [31], and the place fields become more sharply tuned [6].

Although the current model does not describe the formation of place fields themselves, it provides some insight into the process of learning in novel environments. Place fields show considerable plasticity over the course of learning new environments, expanding in adaptation to large environments [32] or over the course of several days of learning (with a concommitant decrease in the number of place cells firing at high rates) [33]. (As presciently noted by Shen et al. [34] in a study of aging rats, the expansion of place fields increases the amount of place field overlap, which can encode more information, at least up to a point.) Furthermore, Karlsson et al. [33] reported that a stable high rate cell population (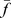
≥25 Hz) emerges over the course of learning a new environment. More specifically, while the overall population firing rate diminishes with learning, the spatial specificity of a small proportion of active cells increases, while neurons that are weakly spatially tuned are suppressed. This is precisely the sort of compensation within *L* predicted by our model: the hippocampus is free to adopt the most efficient parameters within the learning region once a space is learned, and map formation remains stable.

Indeed, perhaps the most striking aspect of the current study is not that it supports the hypothesis that the hippocampus encodes topological information about the environment, but that the learning region *L*, which reflects the scope of biological variability that the hippocampus can afford in a given environment, is rather large. Given the importance of spatial navigation, and thus spatial map formation, to the lives of most animals, it is not surprising that there should be such a wide range of possible firing rates, place field sizes, or cell numbers capable of forming a map of a simple space. (Lose the ability to navigate reliably, and one's lifespan shortens dramatically.) Our model would predict that a degenerating brain that is losing place cells might initially compensate by upregulating firing rate and that such compensation might take place for quite some time before function is noticeably impaired.

Numerous studies have documented spatial learning deficits and changes in place field characteristics in mice bearing specific genetic mutations, but the connection between behavioral changes and the changes in place field properties has been unclear. We suggest that significant alterations of place cell behavior result in hippocampal states hovering at or beyond the boundaries of *L* that cannot consistently support spatial learning. In mouse models of Alzheimer disease (AD), for example, the place fields are larger (less spatially specific), the firing rates lower, and the number of active cells smaller [35], [36]. We speculate that the hippocampal map in AD does not do its job because the parameters of place field activity fall outside the core of the learning region *L_bio_* and therefore cannot reliably encode spatial information. Similarly, acute ethanol intoxication causes place fields to lose their specificity temporarily suppresses place cell firing rate in a dose-dependent manner [37], and the place fields concomittantly lose their spatial specificity [38]; according to our proposed model, the lowest doses of ethanol do not compromise the rat's navigational ability because they allow the place cells still to operate within the learning region. Our model could thus help shed light not only on the process of learning in novel environments, but also on how such abilities can be lost.

## Methods

We open this section by outlining the assumptions we made about place fields and place cells in this first attempt at a model of hippocampal spatial map formation. We then define key theoretical concepts from algebraic topology that motivated our particular computational approach, particularly relating to the relatively new tools of Persistent Homology theory.

### The three environments and simulations of rat trajectories

Each experimental environment depicted in the top row of Figure 4 is 2 meters square. The hole in scenario A is 50*cm *× 50*cm*; the two holes in scenario B are 50*cm *× 50*cm* and 50*cm *× 1*m*, respectively; both holes in scenario C are 180*cm *× 80*cm*. We simulated rat movement to have a mean speed of 25 *cm/sec* (with a range from 0cm/*sec* to 50*cm/sec*) and designed the trajectories to mimic how a rat moves in actual open field experiments: moment by moment, the animal's head changes position by some amount Δ*φ_sim_*, and we reconstructed the histogram of the Δ*φ_exp_* distributions from recorded trajectories so they match the bimodal distribution as found in [39].

### Simulating place cell firing

For this initial analysis, we ignored the details of the spike train structure, such as spike bursting [18] and phase precession [19], and used the simplest cell firing model based on the time rescaling theorem (modeling spiking as “Poisson noise”) [40]. In this approach, place cell firing is represented by an inhomogeneous Poisson process with a time-independent rate function 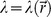, which is a function of the animal's position, 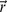 and which produces stochastic firing around place field centers. These Gaussian place fields are not characterized by sharply defined boundaries. Our model thus allows for noise from “erroneous spikes” that may connect place fields in one case and not connect them in another. In the simplest case that is commonly used for place cell activity modeling (cf. [3]), the firing rate *λ_i_* of an individual cell *c_i_* is modulated by a single peak 2*D* Gaussian function,
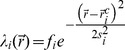
centered around the point 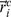 (the center of the *i*th place field) with the variance, 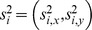.

### Assumptions regarding place fields

In this model, we assume that: 1) Place fields are ellipsoid and omni-directional, as typically recorded in open field environments [41] and are derived from a 2*D* Gaussian rate function (see below.) 2) Place fields within each given simulation are stable, i.e., *P(s)*, the probability distribution of place fields does not change over time. 3) The place field sizes vary according to a log-normal distribution, the center being *s*-mean (cf. Fig. 2). Because our simulations varied both the number of place cells and the size and shape of place fields, we expect that some combinations of parameters tested—e.g., a combination of low cell number N and small mean place field size *s*—will fail to cover the experimental space, while a large N should cover the area uniformly. Indeed, we found that low N and *s*-mean produced high rates of map formation failure (see Figs. 4 and 5).

### Simplicial complexes

Simplicial complexes are used to approximate the structure of topological spaces [42]. For example, a tetrahedron, as a simplicial complex, consists of four triangular facets, six linear edges, and four points in Euclidean space. Each one of these elements is by itself a smaller simplex; this hierarchy is captured in the notion of an abstract simplicial complex, in which the tetrahedron is thought of simply as a set of 4 elements, and any of its 3-element subsets corresponds to a facet, any subset of two corresponds to a segment or edge, and any subset of one corresponds to a vertex. Therefore, given a set of vertices *V*, a *k*-simplex is an unordered subset {*v_0_*, *v_1_*,…, *v_k_*}, where *v_i_*≠*v_j_* for all *i≠j*. The facets of this *k*-simplex consist of all (*k−1*)-simplices of the form *v_0_*, *v_1_*,…, *v_i−1_*, *v_i+1_,…,v_k_*, for some *0≤i≤k*. Geometrically, the *k*-simplex can be described as follows: given *k+1* points in Euclidean space *R^m^* (*m≥k*), the *k*-simplex is a convex body bounded by the union of (*k−1*) linear subspaces of *R^m^* defined by all possible collections of *k* points (chosen out of *k+1* points). Any abstract simplicial complex on a (finite) set of points *V* has a geometric realization in some *R^n^*.

It can be shown that topological features, e.g., holes in the environment, correspond to loops in the simplicial complex, which can be detected through combinatorics of the simplices. It is possible to determine, for example, whether two points in the complex are connected by a sequence of edges or not. The simplicial complex produced by the overlaps between the place fields covering the environment is known in algebraic topology as the “nerve of the cover” or the “nerve simplicial complex” *N(X)* [10], [43], [44].We use the abstract simplicial complex to interpret the pattern of temporal overlaps between the place cell spike trains.

### Homology theory

The hypothesis that drives this project is that the hippocampus encodes a topological map. To begin our investigation we ask whether the topological map produced by the place cells captures the most basic topological features of the environment, namely, the number of holes in it. This question can be addressed using homology theory, which aims to detect homologous loops and to categorize holes in a space. Since the structure of the nerve simplicial complex approximates the structure of the environment, we can use homology theory to count the loops in the simplicial complex and therefore the number of holes in the environment.

There are numerous variants of homology: we use simplicial homology with 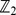 coefficients (the algebraic system consisting of the Boolean values 0 and 1, equipped with “and” as the multiplication and “exclusive or” as addition).

### Betti numbers and homology groups

**Figure 7:**
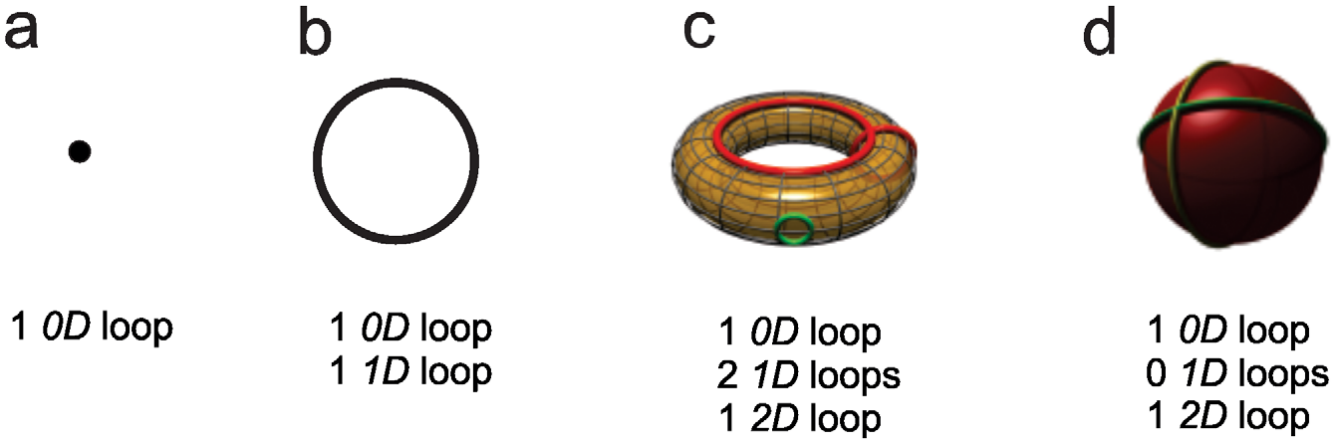
Examples of low-dimensional manifolds and their Betti numbers with some of the corresponding loops. (a) A point is a 0*D* loop; no higher dimensional loops are present. Thus, each manifold containing at least one point has a 0*D* loop, so every list of Betti numbers starts with a “1”. (b) A circle is a 1*D* loop, with no other loops in higher dimensions. (c) A 2*D* torus with two examples of non-contractible (red) 1*D* loops, and an example of a 1*D* loop contractible into a point (green). The 2*D* surface of the torus is the 2*D* loop listed. (d) A 2*D* sphere, with two exemplary contractible *1*D loops. The 2*D* surface of the sphere “loops” onto itself.

Let Σ denote a simplicial complex. Roughly speaking, the homology of Σ, denoted, 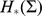 is a sequence of vector spaces *H_k_*(Σ), *k = 0,1,2,…*, where *H_k_*(Σ) is called the *k*-dimensional homology of Σ. The dimension of *H_k_*(Σ), called the *k*th Betti number of Σ, is a coarse measurement of the number of different *k*-dimensional structures, e.g., “loops” in Σ, that cannot be collapsed or deformed into one another (see **Fig. 7**). For example, the simplest basis for *H_0_*(Σ) consists of a choice of vertices, one in each path-component of Σ. Hence the dimension of *H_0_*(Σ) is equal to the number of connected components of Σ. Likewise, the simplest basis for *H_1_*(Σ) consists of looping sequences of 1*D* edges in Σ, which surround *holes* in Σ. For example, if Σ is a 1*D* graph, then the space *H_1_*(Σ) encodes the number and types of loops in the graph.

### Cycles, boundaries and homotopies

For each *k≥0*, let *C_k_(Σ)* be the vector space whose basis is the set of *oriented k*-simplices of Σ; that is, *k*-simplices {*v_0_*,…, *v_k_*} together with an order type denoted [*v_0_*,…, *v_k_*] where a change in orientation corresponds to a change in the sign of the coefficient:
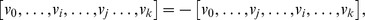
if an odd permutation is used. For *k* larger than the dimension of Σ, we set *C_k_*(Σ) = *0*.

The boundary map is defined to be the linear transformation ∂: *C_k_*→*C_k−1_* which acts on basis elements [*v_0_*,…, *v_k_*] via
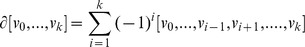
This gives rise to a *chain complex*: a sequence of vector spaces and linear transformations
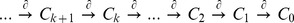
Consider the following two subspaces of *C_k_*: the **cycles** (those subcomplexes without boundary) and the **boundaries** (those subcomplexes which are themselves boundaries) formally defined as:

- *k*-cycles: *Z_k_*(Σ) = Ker(∂: *C_k_*→*C_k−1_*)
- *k*-boundaries: *B_k_*(Σ) = Im(∂: *C_k+1_*→*C_k_*)

A simple lemma demonstrates that ∂ ○ ∂ = 0; that is, the boundary of a chain has an empty boundary. It follows that *B_k_* is a subspace of *Z_k_*. This has significant implications. Just as 1*D* loops on graphs, the *k*-cycles in Σ are the basic objects which count the presence of “holes of dimension *k*” in Σ. Certainly, many of the *k*-cycles in Σ are measuring the same hole; still other cycles do not really detect a hole at all—they bound a subcomplex of dimension *k+1* in Σ. We say that two cycles ζ and *η* in *Z_k_*(Σ) are **homologous** if their difference is a boundary:
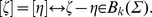
The *k*-dimensional **homology** of Σ, denoted *H_k_*(Σ) is then the quotient vector space
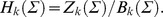
Specifically, an element of *H_k_*(Σ) is an equivalence class of homologous *k*-cycles. This inherits the structure of a vector space in the natural way [*ζ*]+[*η*] = [*ζ+η*] and *c*[*ζ*] = [*cζ*] for 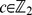. The *k*-th **Betti number** of Σ is then formally defined as the dimension of the *k*-dimensional homology group:
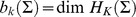
A map *f* : Σ*→Y* is a **homotopy equivalence** if there is a map *g* : *Y→*Σ so that *f ○ g* is homotopic to the identity map on *Y* and *g ○ f* is homotopic to the identity map on Σ. This notion is a weakening of the notion of *homeomorphism*, which requires the existence of a continuous map *g* so that *f ○ g* and *g ○ f* are equal to the corresponding identity maps. The less restrictive notion of homotopy equivalence is useful in understanding relationships between complicated spaces and spaces with simple descriptions.

By arguments utilizing barycentric subdivision, one may show that the homology 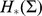 is a **topological invariant** of Σ: it is indeed an invariant of homotopy type. Readers familiar with the Euler characteristic of a triangulated surface will not find it odd that intelligent counting of simplices yields an invariant. For a simple example, the reader is encouraged to contemplate the “physical” meaning of *H_1_*(Σ). Elements of *H_1_*(Σ) are equivalence classes of (finite collections of) oriented cycles in the 1-skeleton of Σ, the equivalence relation being determined by the 2-skeleton of Σ.

### Building simplicial complexes from the spike data: Moving from spatial overlap of place fields to temporal overlap of spike trains

Given a set of place fields {*PF_1_,PF_2_,…,PF_N_*}, with specified shapes and locations, one can use a simple algorithm to construct the simplicial complex N with vertex set {*v_1_*,…,*v_k_*} (one vertex per cell/place field). Two vertices *v_i_* and *v_j_* are connected by an oriented 1*D* bond *σ_ij_* = [*ij*], if the corresponding regions *PF_i_* and *PF_j_* overlap. Three vertices support an oriented 2*D* facet *σ_ijk_* = [*ijk*], if there exists an overlap of three regions *PF_i_*, *PF_j_* and *PF_k_*, and so on. In general, a simplex 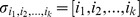 is in N if and only if
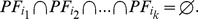
This is the so-called “Čech simplicial complex” or the “*nerve*” complex N [10], [44]. It can be shown that if the set of *PF*s, {*PF_1_,PF_2_,…,PF_N_*}, covers the space *X*,
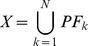
sufficiently densely, then, under fairly general conditions, the nerve complex N has the same homotopy type as the underlying space *X*, and so the topological invariants computed from N will agree with those corresponding to *X* [10], [15], [44]. To be precise, “sufficiently dense” here means that each point of space *X* is contained in at least one place field, and each finite intersection of the fields is contractible.

In the context of studying a hippocampal map formation, in which the analysis is based on temporal characteristics of place cell activity, the simplicial complex can be constructed using the notion of *temporal* overlap between the spike trains rather than *spatial* overlap between place fields. The intuition is the following: If the rat happens to visit the location in space spanned by 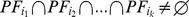, then there is a non-zero probability that the cells 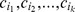 will produce spikes at roughly the same time. Then the coactivity of the place cells can be interpreted as spatial connectivity: if at any a moment of time *t* during the observation period, two neurons *c_i_*, and *c_j_* cofire, then there is a link between the corresponding vertices; if three neurons *c_i_*, *c_j_* and *c_k_* cofire, then there is a 2*D* facet between the vertices and so on. Consider the collection *s_1_*, *s_2_*, …, *s_N_* of spike trains (where each *s_i_* is an ordered list of times at which place cell *c_i_* fires) corresponding to the N cells and fix *ε>0* and 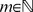. Then we define the simplicial complex *T* by the rule: the simplex


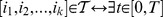
 such that 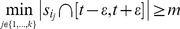.

This defines a “temporal simplicial complex” *T*, which is a direct analogue of the “spatial” simplicial complex N, which summarizes the information contained in the pattern of temporal overlaps between the spike trains and gives a complete topological description of the space *X*.

This construction achieves the goal of providing us with a topological method that can tell us whether cells are indeed receiving all the information necessary for reconstructing the topology of the environment. The main question discussed in the paper is whether and to what extent different hippocampal states (as defined by variations in the mean firing rate 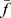, mean place field size 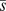, and the number of cells N) affect the network's ability to encode topological information.

In theory, there are two ways in which one can build simplicial complexes in order to describe the topological information contained in place cell firing activity: use place field geometry or place cell spike trains. How are the corresponding simplicial complexes N and *T* related?

It is often remarked that homology is functorial, by which it is meant that it faithfully represents topological information. To clarify this point, consider two simplicial complexes Σ and Σ′. Let f: Σ→Σ′ be a continuous simplicial map: *f* takes each *k*-simplex of Σ to a *k′* -simplex of Σ′, where *k′≤k*. Then, the map f induces a linear transformation *f_#_* : *C_k_*(Σ)→*C_k_*(Σ′). It is a simple lemma to show that *f_#_* takes cycles to cycles and boundaries to boundaries; hence there is a well-defined linear transformation on the quotient spaces
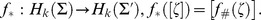
This is called the **induced homomorphism** of *f* on H_*_. Functoriality means that (1) if *f:* Σ*→Y* is continuous then f_*_ : *H_k_*(Σ)→*H_k_*(Y) is a group homomorphism; and (2) the composition of two maps *g ○ f* induces the composition of the linear transformation: (*g ○ f *)_*_ = g_*_ ○ f_*_. This correspondence allows us to not only relate the spatial and the temporal complexes, but to consider the *dynamics* of simplicial complexes used in this paper to study the formation of different of hippocampal maps, using the idea of Homological Persistence.

### Persistent homology and barcodes

Given ε>0 and 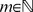 we define a function **f**: *T→R^+^* as follows:
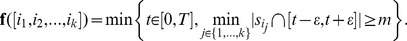
This function is a **filtration** on the simplicial complex *T*, and the pair (*T*, **f**) is called a **filtered simplicial complex**. The concept of filtration is best understood by imagining that the simplicial complex is built across time. One starts with an empty simplicial complex and as time goes by, that is, as the rat explores the environment, the firing of cells “witnesses” [45] the formation of links between the vertices of the simplicial complex. For example, for a cell *i*, by definition **f**(*i*) equals the first time that a significant firing is observed in the spike train *s_i_*. More precisely, **f**(*i*) equals the first time *t* that the spike count for *s_i_* is above *m* in a window centered at *t* of size 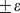.

Note that by definition, for any simplex *σ* containing *i*, **f**(*σ*)≥**f**(*i*). This implies, in particular, that a vertex is added to the simplicial complex earlier than any edge containing the vertex. More generally, one also sees that **f**(*σ*)≥**f**(*τ*) for any 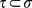. Thus we have an increasing family of simplicial complexes, parameterized by the real line. Indeed, for each *t≥0* let
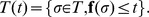
Then, if *t_1_≤t_2_≤…≤t_n_* are all the different values taken by **f**(*σ*) as *σ* ranges in *T*, we have the increasing sequence of simplicial complexes
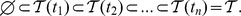
The simplicial complex *T* (*t_n_*) above is the one that could be regarded as a proxy for N: it contains all the connectivity information produced by all the co-firings that occurred before *t_n_*.

Edelsbrunner and colleagues, however, made the following observation [46]: given *t≤t′* there is a natural inclusion of simplicial complexes 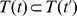. Because of the functoriality property described above, this induces a linear transformation *H_k_*(*T* (*t*))→*H_k_*(*T* (*t′*)) for any *k*. What Edelsbrunner *et al.* observed was that in order to study the homology of a given space one should keep track of the entire system of vector spaces *H_k_*(*T* (*t*)) along with all the linear transformations described above.

Such a system is called a **persistence vector space**. Importantly, it was shown that persistence vector spaces admit a classification analogous to the classification result for finite dimensional vector spaces [47], which asserts that two vector spaces of the same dimension are isomorphic. In the case of persistence vector spaces, it turns out that attached to each is a barcode (see above and Fig. 3). The barcode corresponds to the persistent cycles in the simplicial complex, and any two isomorphic persistence vector spaces have the same barcodes. In the case of the temporal simplicial complex *T*, these barcodes can be interpreted as the “time lines” traced by the topological loops, which characterize the stability of the topological structure defined by place cell co-activity patterns.

To analyze both simulated and experimental data we used jPLEX, a collection of MATLAB functions for computational topology that implements the concepts described above. It is freely available from http://math.stanford.edu/comptop/programs/.

## Supporting Information

Figure S1 **2D slices of point cloud data in** **Figure 4** **(environment A, fourth row), with steady variation in mean firing rate.** Colors and sizes of dots code for the same meanings as described in the legend of Fig. 4: the large blue dots represent the most successful hippocampal states for map formation, with the most rapid map formation times. Here the graphs show a gradual increase in mean firing rate (from 5–31 Hz) and how this affects the overall shape of the learning region. At low firing rates (upper left panels) there is no successful map formation; at 8 Hz, we begin to see some map formation occurring at the largest place field sizes (80–90 cm), especially as the number of neurons increases to 300–350. By 17 and 20 Hz (lower left panels), there is fairly good and rapid map formation with place field sizes around 60 cm. By the time the firing becomes very rapid (31 Hz), smaller place field sizes of 20 cm are able to produce topologically accurate maps, sometimes, but map formation time is long (red dots) so the process is not very efficient.
(TIF)

Figure S2 **2D slices of point cloud data in** **Figure 4** **, (environment A, fourth row) with steady variation in mean place field size.** From these data it appears that the hippocampal state is less sensitive to the chosen range of mean place field sizes (especially between 50 and 80 cm) than it is to firing rate. The graphs show that at this mid-range of place field size, map formation is rapid and accurate (lots of blue dots) for a fairly wide range of firing rates and number of cells in the ensemble.
(TIF)
