# Supplementary figures and images for: A Topological Paradigm for Hippocampal Spatial Map Formation Using Persistent Homology
Source: PLoS Comput Biol. 2012 Aug 9;8(8):e1002581. doi: 10.1371/journal.pcbi.1002581 (PMC3415417; doi:10.1371/journal.pcbi.1002581)

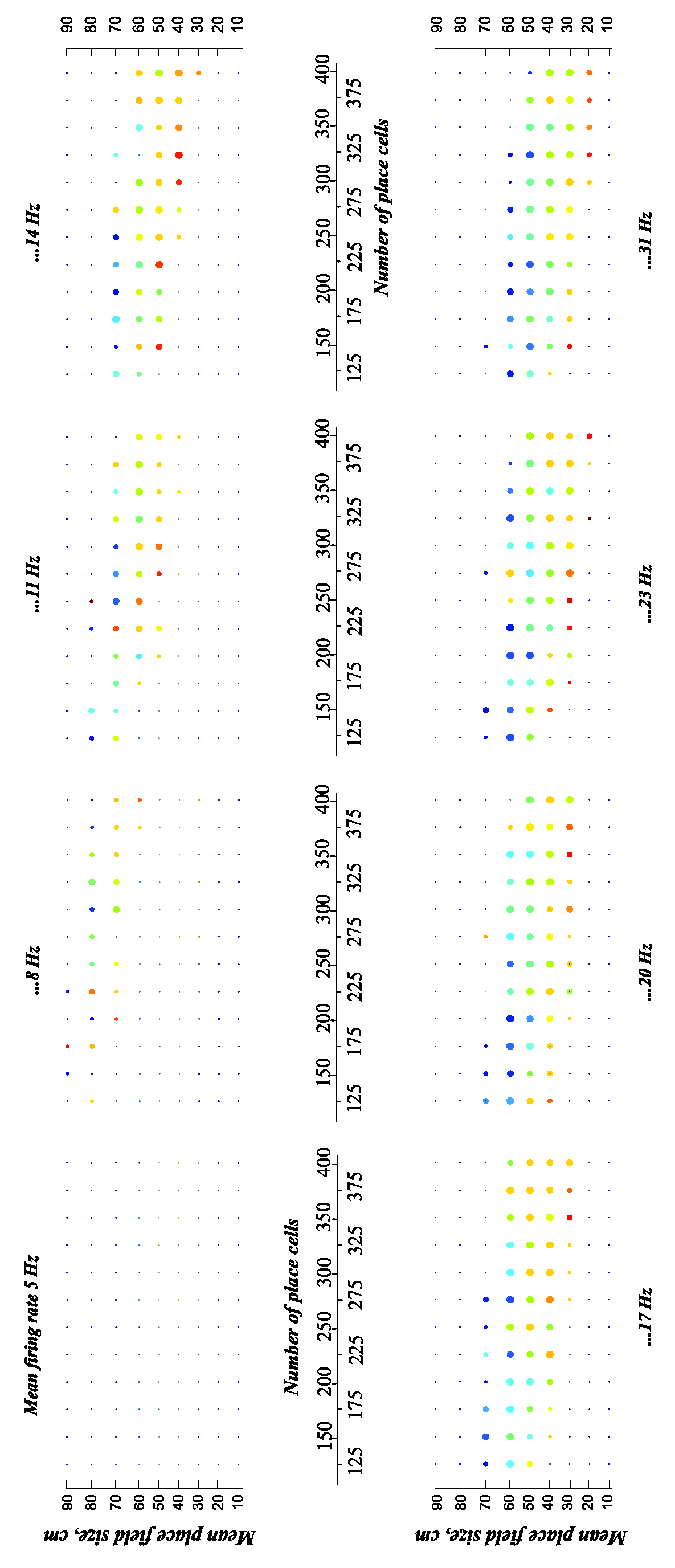

Supplement: Figure S1 — 2D slices of point cloud data in Figure 4 (environment A, fourth row), with steady variation in mean firing rate. Colors and sizes of dots code for the same meanings as described in the legend of Fig. 4: the large blue dots represent the most successful hippocampal states for map formation, with the most rapid map formation times. Here the graphs show a gradual increase in mean firing rate (from 5–31 Hz) and how this affects the overall shape of the learning region. At low firing rates (upper left panels) there is no successful map formation; at 8 Hz, we begin to see some map formation occurring at the largest place field sizes (80–90 cm), especially as the number of neurons increases to 300–350. By 17 and 20 Hz (lower left panels), there is fairly good and rapid map formation with place field sizes around 60 cm. By the time the firing becomes very rapid (31 Hz), smaller place field sizes of 20 cm are able to produce topologically accurate maps, sometimes, but map formation time is long (red dots) so the process is not very efficient. (TIF) [file pcbi.1002581.s001.tif]

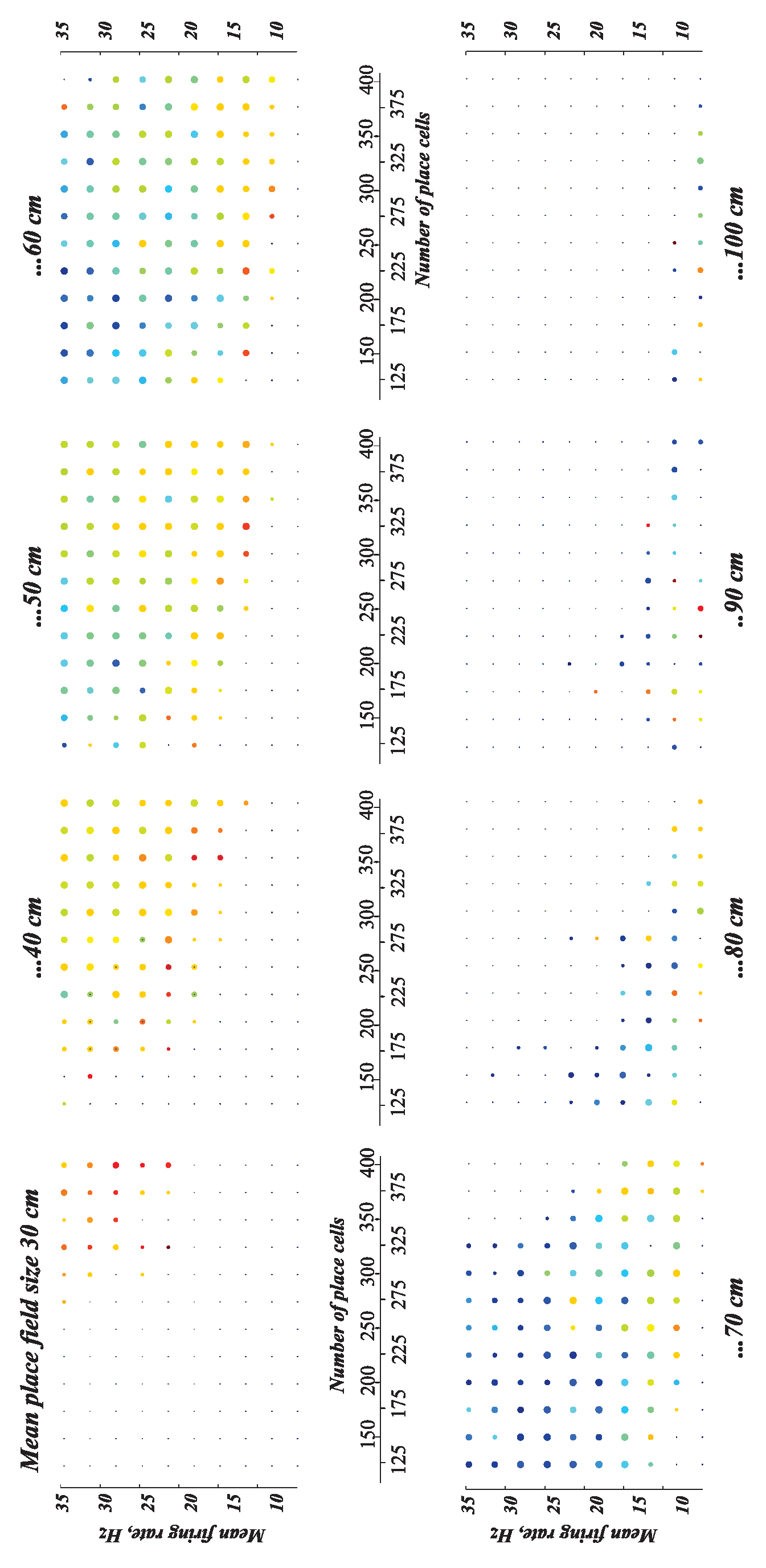

Supplement: Figure S2 — 2D slices of point cloud data in Figure 4 , (environment A, fourth row) with steady variation in mean place field size. From these data it appears that the hippocampal state is less sensitive to the chosen range of mean place field sizes (especially between 50 and 80 cm) than it is to firing rate. The graphs show that at this mid-range of place field size, map formation is rapid and accurate (lots of blue dots) for a fairly wide range of firing rates and number of cells in the ensemble. (TIF) [file pcbi.1002581.s002.tif]
